# Silencing of lncRNA EZR-AS1 inhibits proliferation, invasion, and migration of colorectal cancer cells through blocking transforming growth factor β signaling

**DOI:** 10.1042/BSR20191199

**Published:** 2019-11-13

**Authors:** Zhenhua Liu, Ning Wang, Feiqing Wang, Shuaimin Zhang, Jie Ding

**Affiliations:** 1 Medical College of Guizhou University, No. 2708, South Section of Huaxi Avenue, Huaxi District, Guiyang City, Guizhou Province 550025, China; 2 Department of Hepatobiliary Surgery, Guizhou Provincial People’s Hospital, No. 83, Zhongshan East Road, Guiyang City, Guizhou Province 550002, China; 3 Department of Pharmacy, Guizhou Orthopaedic Hospital, No. 123, Shachong South Road, Guiyang City, Guizhou Province 550002, China; 4 Department of Clinical Laboratory, The First Affiliated Hospital of Guizhou University of Traditional Chinese Medicine, No. 71, Baoshan North Road, Guiyang City, Guizhou Province 550001, China; 5 Department of Gastrointestinal Surgery, Guizhou Provincial People’s Hospital, No. 83, Zhongshan East Road, Guiyang City, Guizhou Province 550002, China

**Keywords:** Colorectal cancer, Long non-coding RNA EZR-AS1, Migration, Proliferation, Transforming growth factor β

## Abstract

Long non-coding RNA (lncRNA) plays a key regulatory role in the pathogenesis of colorectal cancer (CRC). In the present study, the specific regulatory role of lncRNA ezrin antisense RNA 1 (EZR-AS1) on CRC was investigated. The expression of lncRNA EZR-AS1 was significantly up-regulated in CRC cell lines (HCT8, HCT116, HT29, and SW620 cells), which was significantly different from that of normal human fetal colonic mucosa cells (FHC cells) (*P*<0.01). HCT116 and HT29 cells were then transfected with EZR-AS1 shRNA (sh-EZR-AS1) to silence lncRNA EZR-AS1 (*P*<0.01). When compared with the Control, after transfection of SH-EZR-AS1, E-cadherin was up-regulated, Vimentin was down-regulated, the apoptosis rate was increased, the cell viability, wound healing rate, and the number of invasive cells were decreased in HCT116 and HT29 cells (*P*<0.05). Silencing of lncRNA EZR-AS also significantly reduced the tumor volume and weight in mice injected with sh-EZR-AS1-transfected HCT116 and HT29 cells (*P*<0.05). The regulatory relationship between lncRNA EZR-AS1 and transforming growth factor β (TGF-β) signaling was further identified in CRC cells. Silencing of lncRNA EZR-AS1 significantly down-regulated TGF-β, Smad2, and α-SMA expression in HCT116 and HT29 cells at the protein level (*P*<0.05). The intervention of SB431542 (a TGF-β receptor blocker) and silencing of Smad2 both significantly down-regulated lncRNA EZR-AS1 expression in HCT116 and HT29 cells (*P*<0.01). In conclusion, silencing of lncRNA EZR-AS1 inhibited the proliferation, invasion, migration, and epithelial–mesenchymal transition, and promoted the apoptosis of CRC cells through blocking TGF-β signaling.

## Introduction

Colorectal cancer (CRC) is the third most common cancer and the fourth leading cause of cancer-related death in the world [1]. Colorectal cancer mainly occurs in colon or rectum, and is related to many risk factors, such as age, diet habit, and chronic disease history [2]. In clinical practice, surgery, chemotherapy, and radiotherapy are still the main therapeutic strategies for CRC [3,4]. However, the prognosis of CRC patients remains poor, especially for those with metastatic CRC [5]. The discovery of novel therapeutic targets for CRC is urgently needed.

Long non-coding RNAs (lncRNAs) are a class of non-coding RNAs longer than 200 nucleotides [6]. A previous study has demonstrated that some lncRNAs are up-regulated in CRC, such as lncRNA H19, HOX transcript antisense RNA (HOTAIR), metastasis associated lung adenocarcinoma transcript 1 (MALAT1), hepatocellular carcinoma up-regulated long non-coding RNA (HULC), colon cancer associated transcript 1/2 (CCAT1/2), colorectal neoplasia differentially expressed (CRNDE), myosin light chain kinase pseudogene 1 (MYLKP1), taurine up-regulated 1 (TUG1), and urothelial carcinoma associated 1 (UCA1), and some are down-regulated in CRC, such as lncRNA maternally expressed 3 (MEG3), P21, phosphatase and tensin homolog pseudogene 1 (PTENP1), and low expression in tumor (LET) [2]. These lncRNAs play different regulatory roles in the pathogenesis of CRC. For examples, the expression of UCA1 is positively associated with the tumor size and depth, histological grade, and poor prognosis in CRC patients [7]. Overexpression of MALAT1 and TUG1 promotes the proliferation and migration of CRC cells *in vitro*, as well as the tumor growth and metastasis in mice [8,9]. The expression of MEG3 is negatively associated with the histological grade, tumor invasion, and tumor node metastasis stage of CRC patients, and overexpression of MEG3 inhibits the proliferation of CRC cells *in vitro* and *in vivo* [10]. Long non-coding RNA ezrin antisense RNA 1 (EZR-AS1) is a natural antisense lncRNA transcribed from the opposite strand in the EZR gene locus [11]. A previous study has demonstrated that knockdown of EZR-AS1 significantly inhibits the migration of esophageal squamous cell carcinoma (ESCC) cells, reduces the tumor volume and weight, and reduces the number of metastatic lymph nodules in mice [11]. However, the specific regulatory role and mechanism of lncRNA EZR-AS1 on CRC remain unclear.

The regulatory effect of lncRNA in CRC is closely related to the regulation of a variety molecular signalings, such as phosphoinositide 3-kinase (PI3K)/Akt [12], Wnt/β-catenin [13], nuclear factor-kappa B (NF-κB) [14], and transforming growth factor β (TGF-β)/Smad [15]. Transforming growth factor β signaling plays a key regulatory role in diverse cellular processes of CRC, including cell proliferation, apoptosis, invasion, and migration [16,17]. Previous studies have demonstrated that blocking of TGF-β signaling contributes to the anti-tumor effects of various lncRNAs, such as lncRNA antisense non-coding RNA in the INK4 locus (ANRIL) [18], inactive X specific transcripts (XIST) [19], activated by TGF-β (ATB) [20], TGF-β induced lncRNA (TBILA) [21], and Smad7 [22]. However, the regulatory relationship between lncRNA EZR-AS1 and TGF-β signaling remains unclear. In the present study, the expression of lncRNA EZR-AS1 was detected in CRC cells. The specific regulatory role of lncRNA EZR-AS1 on the proliferation, apoptosis, invasion, migration, and epithelial–mesenchymal transition (EMT) of CRC cells were evaluated by silencing lncRNA EZR-AS1. The potential regulatory relationship between lncRNA EZR-AS1 and TGF-β signaling was further analyzed. Our findings may provide a novel therapeutic target for CRC and open up new insights into the underlying mechanism.

## Methods

### Cell culture

Four human CRC cell lines, HCT8, HCT116, HT29, and SW620, which are with different origins and genetic characteristics, and FHC, a normal human fetal colonic mucosa cell line were purchased from the Cell Bank of the Chinese Academy of Science (Shanghai, China). All cells were maintained in Roswell Park Memorial Institute (RPMI) 1640 medium (HyClon, Loga, UT, U.S.A.) containing 10% fetal bovine serum (FBS), and cultured at 37°C with 5% CO_2_ in a constant temperature incubator (MCO-15AC, SANYO, Osaka, Japan). The medium was refreshed every two days, and cells were passaged until 90% confluence. Logarithmic growth phase cells were used for further assays.

### Cell treatments

The shRNAs of EZR-AS1 (sh-EZR-AS1), EZR-AS1 negative control (NC-EZR-AS1), Smad2 (sh-Smad2), and Smad2 negative control (NC-Smad2) were purchased from Shanghai Jima Pharmaceutical Technology Co., Ltd. (Shanghai, China). Cells were cultured until 80% confluence and transfected with the specific shRNAs using Lipofectamine 2000 (Invitrogen, Carlsbad, CA, U.S.A.) for 48 h. In addition, HCT116 and HT29 cells were treated with 10 ng/ml TGF-β (Sigma, St. Louis, MO, U.S.A.), and TGF-β combined with 10 uM SB431542 (a TGF-β receptor blocker) (Sigma) for 72 h, Cells were used for further assays after the treatments.

### Quantitative real-time PCR

Total RNA was extracted from cells using TRIzol agent (Invitrogen), and reverse-transcribed into cDNA using a cDNA Reverse Transcription Kit (Invitrogen) in accordance with manufacturers’ instructions. Quantitative real-time PCR (qRT-PCR) was performed on ABI 7500 (ABI, Foster City, CA, U.S.A.) by using specific primers (lncRNA EZR-AS1 F: 5′-CCCTCTCCAATGAAGCCTCTC-3′, R: 5′-ACCGAA AATGCCGAAACCAG-3′; EZR-F: 5′-CTTTTGGGAGCGCGGGCAGC-3′, R: 5′-AGACGCTGTCCCAACCCGGC-3′). Glyceraldehyde-3-phosphate dehydrogenase (GAPDH) was used as an internal control (GAPDH F: 5′-TCCTCTGACTTCAACAGCGACAC-3′, R: 5′-CACCCTGTTGCTGTAGCCAAATTC-3′). The PCR program included 95°C for 10 min, 50 cycles at 95°C for 15 s, 60°C for 1 min, and 72°C for 40 s. Relative expression of lncRNA EZR-AS1 was calculated according to the 2^-ΔΔ*C*_t_^ method [23]. This assay was repeated five times for each gene.

### MTT assay

Cell viability was detected by MTT assay. Briefly, cells at a volume of 200 μl were seeded in 96-well plates at a density of 6 × 10^3^ per well, and then incubated with 20 μl MTT (Sigma, St. Louis, MO, U.S.A.) for 4 h. The medium was then removed and cells were incubated with 150 μl dimethyl sulfoxide (DMSO) for 10 min. Optical density (OD) at 495 nm was detected by a Microplate Reader (Invitrogen). This assay was repeated three times for each group.

### Cell apoptosis assay

Cell apoptosis was detected by using Annexin V-propidium iodide (PI) kit (Invitrogen) in accordance with manufacturer’s instruction. Briefly, cells were re-suspended in binding buffer at a density of 3 × 10^5^ per well, and then stained with Annexin V-Enhanced green fluorescent protein and PI on ice under darkness for 10 min. The apoptosis rate was analyzed on a Flow Cytometry (BD, Franklin Lakes, NJ, US.A.). This assay was repeated three times for each group.

### Transwell assay

Invasion ability of cells was detected by using transwell cell culture chambers (BD). Briefly, 100 μl cells were seeded in the upper chamber (pre-coated with Matrigel) at a density of 1×10^3^ per μl. The lower chamber was filled with 500 µl RPMI 1640 medium containing 10% FBS. After 48 h of incubation at 37°C, cells in the upper chamber were removed. Cells in the lower chamber were fixed in 4% formaldehyde for 15 min, stained with 0.1% crystal violet. Positive stained cells were counted in five randomly selected fields under microscope (Olympus, Tokyo, Japan). This assay was repeated three times for each group.

### Wound healing assay

Migration ability of cells was detected by Wound healing assay. Briefly, cells were seeded in 6-well plates at a density of 5 × 10^5^ per well. When 90% confluence was achieved, a wound track was scored in each plate with a plastic scraper. The cell debris was removed by washing with PBS. After 48 h of culturing, the migration distance was measured under microscope (Olympus). This assay was repeated three times for each group.

### Western blot

Cells were lysed in RIPA Lysis buffer (Invitrogen). Total proteins were separated by 10% sodium dodecyl sulfate–polyacrylamide gel electrophoresis, and transferred to polyvinylidenefluoride membrane (Millipore, Billerica, MA, U.S.A.). The membrane was blocked with 5% skim milk in Tris-buffered saline Tween (TBST) for 1 h, and incubated overnight at 4°C with specific primary antibodies, including anti-Smad2, anti-phosphorylated Smad2 (anti-pSmad2), anti-E-cadherin, anti-N-cadherin, anti-TGF-β, anti-α smooth muscle actin (anti-α-SMA), and anti-GAPDH (Rabbit anti-human 1:100, Abcam, Cambridge, MA, U.S.A.). After being washed with TBST for three times, the membrane was incubated at 25°C for 1 h with horseradish peroxidase (HRP)-conjugated secondary antibody (goat anti-rabbit, 1:1000, Abcam). The protein bands were visualized using HRP color development kit (Invitrogen). This assay was repeated for three times each protein.

### Establishment of tumor model in mice

Forty-eight mice were purchased from Shanghai Laboratory Animal Center, Chinese Academy of Sciences (Shanghai, China). Mice were fed in an animal room at 22°C and allowed free access to water and food. HCT116 and HT29 cells at a volume of 200 ml in different groups (sh-EZR-AS1, NC-EZR-AS1, and Control) were injected into the right axilla of mice at a density of 0.1×10^8^ cells/ml. The tumor volume was measured with vernier caliper every 1 week (*N* = 16 in each group). Mice were killed by cervical dislocation after the injection for 5 weeks, and the tumor weight was measured by using analytical balance (*N* = 16 in each group). Animal experiments were conducted after obtaining approval of Guizhou Provincial People’s Hospital’s ethical committee (NO. EC Review-Animal-2019-008). All the animal experiments were conducted in the Science Building of Guizhou Provincial People’s Hospital.

### Statistical analyses

All data were expressed as mean ± standard deviation (SD). Statistical analysis was performed by SPSS version 18.0 (SPSS Inc., Chicago, IL, U.S.A.). Comparison between different groups was determined by LSD *t*-test (two groups) or one-way ANOVA followed by *post hoc* test (more than two groups). A *P*-value less than 0.05 was considered as statistically significant.

## Results

### Long non-coding RNA EZR-AS1 was up-regulated in CRC cells

**Figure 1:**
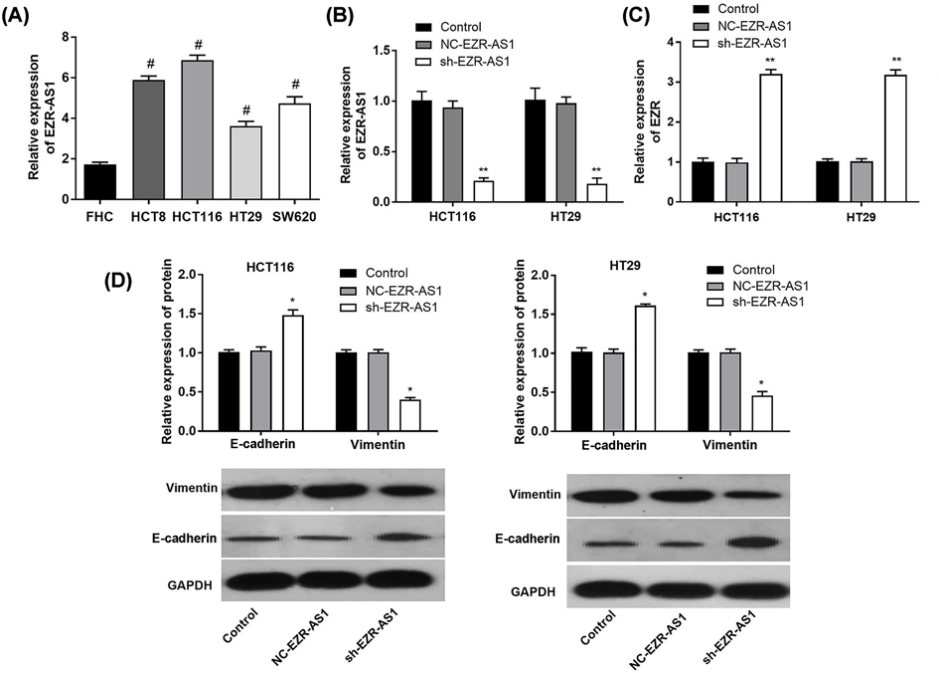
The expression of lncRNA EZR-AS1, E-cadherin, and Vimentin in colorectal cancer cells (**A**) Relative expression of lncRNA EZR-AS1 in four CRC cell lines, HCT8, HCT116, HT29, and SW620, and a normal human fetal colonic mucosa cell line FHC (mRNA level) (*N* = 5). (**B**) Relative expression of lncRNA EZR-AS1 in transfected HCT116 and HT29 cells (mRNA level) (*N* = 5). (**C**) Relative expression of Ezrin (EZR) in transfected HCT116 and HT29 cells (mRNA level) (*N* = 5). (**D**) Relative expression of E-cadherin and Vimentin in transfected HCT116 and HT29 cells (protein level) (*N* = 3). Control, normal cells without treatment; NC-EZR-AS1, cells transfected with EZR-AS1 shRNA negative control; sh-EZR-AS1, cells transfected with EZR-AS1 shRNA. ^#^*P*<0.05 vs. FHC; *P<0.05; **P<0.01 vs. Control and NC-EZR-AS1.

Compared with FHC cells, the expression of lncRNA EZR-AS1 in CRC cell lines HCT8, HCT116, HT29, and SW620 cells was significantly up-regulated at the mRNA level (*P*<0.05) (Figure 1A). Then, HCT116 cells with relatively high lncRNA EZR-AS expression, and HT29 cells with relatively low lncRNA EZR-AS1 expression were silenced by sh-EZR-AS1 transfection. The transfection of sh-EZR-AS1 significantly down-regulated lncRNA EZR-AS1 expression in HCT116 and HT29 cells at the mRNA level (*P*<0.01) (Figure 1B). In addition, the transfection of sh-EZR-AS1 significantly up-regulated EZR expression in HCT116 and HT29 cells at the mRNA level (*P*<0.01) (Figure 1C). The expression of lncRNA EZR-AS1 and EZR was not significantly influenced by the transfection of NC-EZR-AS1 in HCT116 and HT29 cells (Figure 1B,C).

### Silencing of lncRNA EZR-AS1 inhibited the EMT of HCT116 and HT29 cells

As shown in Figure 1C, silencing of lncRNA EZR-AS1 significantly up-regulated E-cadherin expression, and down-regulated Vimentin expression in HCT116 and HT29 cells at the protein level (*P*<0.05). The protein levels of E-cadherin and Vimentin in HCT116 and HT29 cells were not significantly influenced by silencing of lncRNA EZR-AS1 (Figure 1D).

### Silencing of lncRNA EZR-AS1 inhibited the proliferation, and promoted the apoptosis of HCT116 and HT29 cells

**Figure 2:**
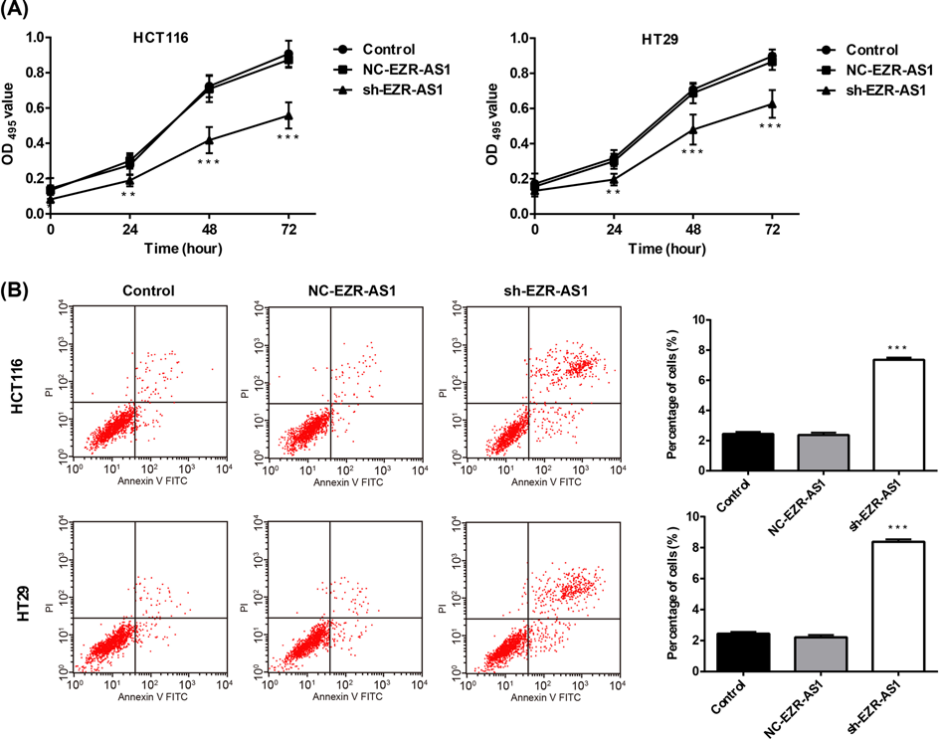
The proliferation and apoptosis of colorectal cancer cells (**A**) The viability of HCT116 and HT29 cells (OD_495_ value) (*N* = 3). (**B**) The apoptosis rate of HCT116 and HT29 cells (*N* = 3). Control, normal cells without treatment; NC-EZR-AS1, cells transfected with EZR-AS1 shRNA negative control; sh-EZR-AS1, cells transfected with EZR-AS1 shRNA. ***P<0.001 vs. Control and NC-EZR-AS1.

The cell viability (OD_495_ value) was significantly increased in HCT116 and HT29 cells with culture time in a time-dependent manner. At 24, 48, and 72 h post-culturing, significantly lower cell viability was observed in sh-EZR-AS1-transfected HCT116 and HT29 cells than in the Control (*P*<0.01) (Figure 2A). At 48 h post-transfection, the apoptosis rate was significantly higher in sh-EZR-AS1-transfected HCT116 and HT29 cells than in the Control (*P*<0.01) (Figure 2B). The proliferation and apoptosis of HCT116 and HT29 cells were not significantly influenced by the transfection of NC-EZR-AS1 (Figure 2A,B).

### Silencing of lncRNA EZR-AS1 inhibited the migration and invasion of HCT116 and HT29 cells

**Figure 3:**
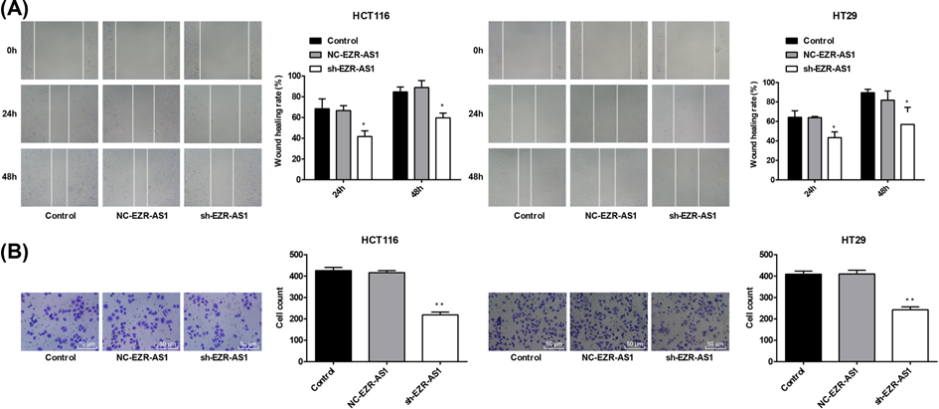
The migration and invasion of colorectal cancer cells (**A**) Wound healing rate of HCT116 and HT29 cells (*N* = 3). (**B**) The number of invasive HCT116 and HT29 cells (*N* = 3). Control, normal cells without treatment; NC-EZR-AS1, cells transfected with EZR-AS1 shRNA negative control; sh-EZR-AS1, cells transfected with EZR-AS1 shRNA. *P<0.05; **P<0.01 vs. Control and NC-EZR-AS1.

Significantly lower wound healing rate was observed in sh-EZR-AS1-transfected HCT116 and HT29 cells than in the Control (*P*<0.05) (Figure 3A). In addition, the number of invasive cells was significantly lower in sh-EZR-AS1-transfected HCT116 and HT29 cells than in the Control (*P*<0.05) (Figure 3B). The migration and invasion of HCT116 and HT29 cells were not significantly influenced by the transfection of NC-EZR-AS1 (Figure 3A,B).

### Silencing of lncRNA EZR-AS1 inhibited the tumor growth in mice

**Figure 4:**
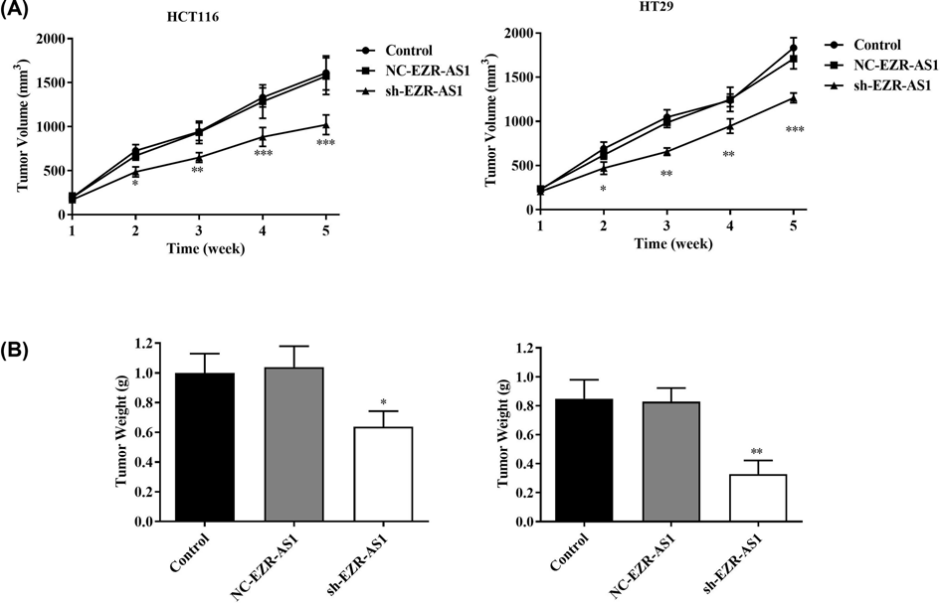
The tumor growth in mice injected with colorectal cancer cells (**A**) The tumor volume in mice injected with HCT116 and HT29 cells every one week (*N* = 16 in each group). (**B**) The tumor weight in mice injected with HCT116 and HT29 cells for 5 weeks (*N* = 16 in each group). Control, mice injected with normal cells without treatment; NC-EZR-AS1, mice injected with EZR-AS1 shRNA negative control-transfected cells; sh-EZR-AS1, mice injected with EZR-AS1 shRNA-transfected cells. *P<0.05; **P<0.01; ***P<0.001 vs. Control and NC-EZR-AS1.

The tumor volume in mice injected with HCT116 and HT29 cells was significantly increased in a time-dependent manner. Starting from the second week, the tumor volume in mice injected with sh-EZR-AS1-transfected HCT116 and HT29 cells was significantly lower than that in the Control (*P*<0.05) (Figure 4A). Five weeks after injection, the tumor weight was significantly lower in mice injected with sh-EZR-AS1-transfected HCT116 and HT29 cells than that in the Control (*P*<0.05). Both the tumor volume and weight were not significantly influenced by the transfection of NC-EZR-AS1 (Figure 4A,B).

### Silencing of lncRNA EZR-AS1 blocked TGF-β signaling in HCT116 and HT29 cells

**Figure 5:**
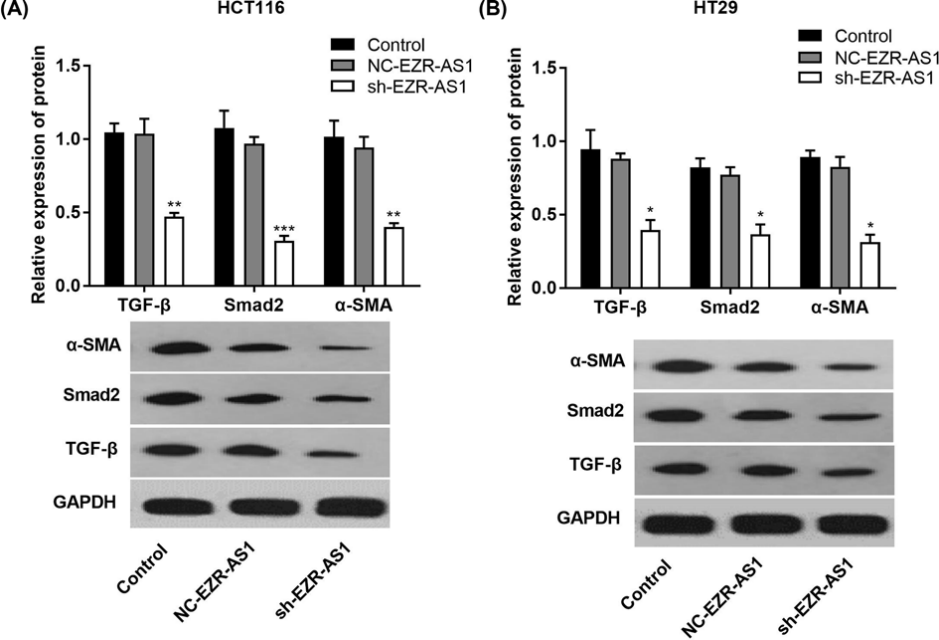
The TGF-β signaling in colorectal cancer cells The expression of transforming growth factor β (TGF-β), Smad2, and α smooth muscle actin (α-SMA) in HCT116 (**A**) and HT29 (**B**) cells at the protein level (*N* = 3). Control, normal cells without treatment; NC-EZR-AS1, cells transfected with EZR-AS1 shRNA negative control; sh-EZR-AS1, cells transfected with EZR-AS1 shRNA. *P<0.05; **P<0.01; ***P<0.001 vs. Control and NC-EZR-AS1.

Silencing of lncRNA EZR-AS1 significantly down-regulated the expression of TGF-β, Smad2 (a signal transduction protein of TGF-β), and α-SMA (a response protein of TGF-β) in HCT116 and HT29 cells at the protein level (*P*<0.01). The protein levels of TGF-β, Smad2, and α-SMA in HCT116 and HT29 cells were not significantly influenced by the transfection of NC-EZR-AS1 (Figure 5A,B).

### Blocking of TGF-β signaling down-regulated lncRNA EZR-AS1 in HCT116 and HT29 cells

**Figure 6:**
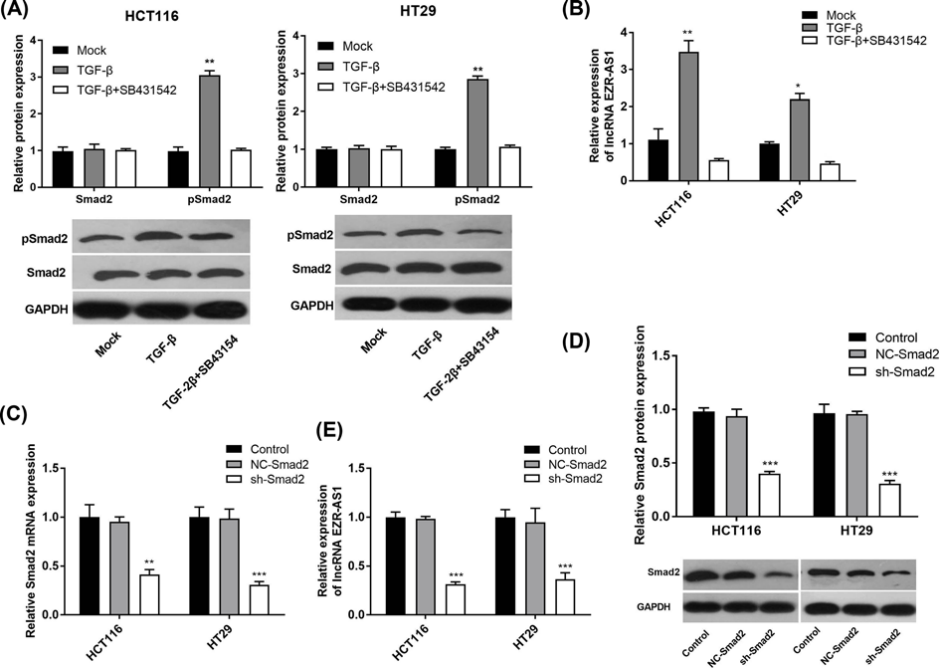
The expression of Smad2, phosphorylated Smad2 (pSmad2) and long non-coding RNA (lncRNA) EZR-AS1 in colorectal cancer cells (**A**) Relative expression of Smad2 and pSmad2 in HCT116 cells and HT29 cells (protein level) (*N* = 3). (**B**) Relative expression of lncRNA EZR-AS1 in HCT116 and HT29 cells (mRNA level) (*N* = 5). Mock, normal cells without treatment; TGF-β, cells treated with 10 ng/ml TGF-β; TGF-β + SB431542, cells treated with 10 ng/ml TGF-β and 10 µM SB431542. *P<0.05; **P<0.01 vs. Mock and TGF-β + SB431542. (**C**) Relative expression of Smad2 in HCT116 and HT29 cells (mRNA level) (*N* = 5). (**D**) Relative expression of Smad2 in HCT116 and HT29 cells (protein level) (*N* = 3). (**E**) Relative expression of lncRNA EZR-AS1 in HCT116 and HT29 cells (mRNA level) (*N* = 5). Control, normal cells without treatment; NC-Smad2, cells transfected with Smad2 shRNA negative control; sh-Smad2, cells transfected with Smad2 shRNA. *P<0.05; **P<0.01; ***P<0.001 vs. Control and NC-Smad2.

The intervention of SB431542 significantly inhibited TGF-β-induced up-regulation of pSmad2 in HCT116 and HT29 cells at the protein level (*P*<0.01) (Figure 6A), and TGF-β-induced up-regulation of lncRNA EZR-AS1 expression (*P*<0.05) (Figure 6B). In addition, Smad2 was further silenced to block TGF-β signaling in HCT116 and HT29 cells by the transfection of sh-Smad2 (*P*<0.01) (Figure 6C,D). Silencing of Smad2 also significantly down-regulated lncRNA EZR-AS1 expression in HCT116 and HT29 cells (*P*<0.01) (Figure 6E).

## Discussion

Colorectal cancer is a common gastrointestinal malignancy that seriously affects people’s health [24]. Although the treatment of CRC has made encouraging advantages, the prognosis of CRC patients remains poor [25]. With the discovery of molecular mechanisms involved in the pathogenesis of CRC, lncRNAs have been considered as important regulators in the occurrence and development of CRC [26]. Long non-coding RNA EZR-AS1 is a specific lncRNA that up-regulated in both ESCC tissues and cells [11]. However, the expression level of lncRNA EZR-AS1 in CRC remains unclear. In the present study, we found that lncRNA EZR-AS1 expression was significantly up-regulated in CRC cell lines (HCT8, HCT116, HT29, and SW620 cells) than in normal human fetal colonic mucosa cell line (FHC cells). Our findings illustrate that lncRNA EZR-AS1 may act as a tumor promoter in CRC.

Long non-coding RNA plays an important regulatory role in diverse cellular processes, such as cell proliferation, apoptosis, differentiation, and invasion [27]. In the present study, lncRNA EZR-AS1 was silenced in HCT116 and HT29 cells by the transfection of sh-EZR-AS1, and then the effects of lncRNA EZR-AS1 silencing on the proliferation and apoptosis of CRC cells were analyzed. We found that sh-EZR-AS1-transfected HCT116 and HT29 cells exhibited significantly lower cell viability, and higher apoptosis rate than the Control. These findings indicate that silencing of lncRNA EZR-AS1 inhibits the proliferation, and promotes the apoptosis of CRC cells *in vitro*. Previous studies have demonstrated that down-regulation of lncRNA up-regulated in hepatocellular carcinoma (URHC) and forkhead box P4 antisense RNA 1 (FOXP4-AS1) can inhibit the proliferation, and promote the apoptosis of CRC cells [28,29]. Silencing of lncRNA EZR-AS1 may exert similar anti-proliferation role on CRC cells with the down-regulation of lncRNA URHC and FOXP4-AS1. In order to further identify the anti-proliferation effect of lncRNA EZR-AS1 silencing *in vivo*, sh-EZR-AS1-transfected HCT116 and HT29 cells were injected into mice. We found that silencing of lncRNA EZR-AS1 significantly reduced the tumor volume and weight in mice injected with HCT116 and HT29 cells. These findings further indicate that silencing of lncRNA EZR-AS1 also has a significant anti-tumor potential on CRC *in vivo*.

Tumor metastasis is closely related to the poor prognosis of patients with CRC [5]. Previous studies have demonstrated that the invasion and migration ability of CRC cells can be inhibited by the down-regulation of lncRNA Angelman syndrome chromosome region (ANCR) [30], HOTAIR [31], and transcription factor 7 (TCF7) [32]. In the present study, silencing of lncRNA EZR-AS1 exhibited similar anti-migration and anti-invasion effects on CRC cells with the lncRNAs mentioned above. We found that the wound healing rate and the number of invasive cells of sh-EZR-AS1-transfected HCT116 and HT29 cells were significantly lower than those of the Control. In addition, a previous study has demonstrated that knockdown of EZR-AS1 significantly inhibits the migration of ESCC cells [11]. Our findings illustrate that the silencing of lncRNA EZR-AS1 cannot only inhibit the migration, but also the invasion of CRC cells. On the other hand, E-cadherin and Vimentin are known as marker proteins in EMT. A previous study has demonstrated that down-regulation of lncRNA URHC increased the expression of E-cadherin and reduces the expression of Vimentin in LOVO cells [28]. In the present study, we found that silencing of lncRNA EZR-AS1 significantly up-regulated E-cadherin, and down-regulated Vimentin expression in HCT116 and HT29 cells. These findings illustrate that silencing of lncRNA EZR-AS1 inhibits the EMT of HCT116 and HT29 cells. Since EMT is an absolute requirement for tumor metastasis [33], silencing of lncRNA EZR-AS1 may suppress the metastasis of CRC through inhibiting EMT.

Transforming growth factor β signaling is usually activated in CRC, and plays an important role in the development and progression of CRC [16]. A positive interaction between lncRNA ANRIL and TGF-β signaling has been identified in both prostate cancer cells and thyroid cancer cells [15,18]. It has been reported that overexpression of lncRNA ANRIL promotes the proliferation and migration of prostate cancer cells *via* activating TGF-β1/Smad signaling [15]. Silencing of lncRNA ANRIL inhibits the proliferation, invasion, and metastasis of thyroid cancer cells through inhibiting TGF-β/Smad signaling [18]. In the present study, the specific regulatory relationship between lncRNA EZR-AS1 and TGF-β signaling was analyzed. We found that silencing of lncRNA EZR-AS1 significantly down-regulated TGF-β, Smad2, and α-SMA expression in HCT116 and HT29 cells. Since TGF-β, Smad2, and α-SMA are positively related to the activation of TGF-β signaling, our findings illustrate that the silencing of lncRNA EZR-AS1 blocks TGF-β signaling in CRC cells. TGF-β usually acts as an oncogene in CRC cells and overproduction of TGF-β may affect the tumor environment via suppression of tumor-infiltrating immune cells, and contribute to tumor cell aggressiveness through autocrine activation of Smad signaling [34]. TGF-β receptor-induced phosphorylation of Smad2 and Smad3 can bind to Smad4, and Smad complexes may regulate the transcription of a multitude of TGF-β-responsive genes involved in diverse cellular process, such cell proliferation, apoptosis, invasion, and migration [34,35]. Therefore, blocking of TGF-β signaling may directly contribute to the inhibition effects of lncRNA EZR-AS1 silencing on the proliferation, invasion, migration, and EMT of CRC cells. In order to further identify the interaction between lncRNA EZR-AS1 and TGF-β signaling in CRC cells, TGF-β signaling was blocked in CRC cells by the intervention of SB431542 (a TGF-β receptor blocker) or the transfection of sh-Smad2. We found that the intervention of SB431542 and transfection of sh-Smad2 both significantly down-regulated lncRNA EZR-AS1 expression in HCT116 and HT29 cells. These findings indicate that blocking of TGF-β signaling can also down-regulate lncRNA EZR-AS1 in CRC cells in a feedback manner. To sum up, a positive mutual interaction between lncRNA EZR-AS1 and TGF-β signaling is identified on CRC cells.

The present study also has some limitations. First, lncRNA EZR-AS1 expression is positively correlated with EZR expression [11], yet the regulatory relationship between EZR expression and the anti-tumor effect of lncRNA EZR-AS1 silencing on CRC cells is not investigated. Thus many, if not all, the effect of manipulating lncRNA EZR-AS1 can be through changes in EZRIN expression. Second, the effect of lncRNA EZR-AS1 silencing on tumor metastasis is not analyzed *in vivo*. Third, there is limited understanding of the efficacy and safety of lncRNA EZR-AS1 silencing in the treatment of CRC, and limited discovery of lncRNA EZR-AS1-target drugs. Further researches on these fields are urgently needed.

In conclusion, lncRNA EZR-AS1 is an oncogene that up-regulated in CRC cells. Silencing of lncRNA EZR-AS1 inhibits the proliferation, invasion, migration, and EMT, and promotes the apoptosis of CRC cells through blocking TGF-β signaling. Because silencing of EZR-AS1 also inhibits the tumor growth *in vivo* (mice), EZR-AS1 is a promising therapeutic target for CRC.

## Abbreviations

ANRIL: antisense non-coding RNA in the INK4 locus
CRC: colorectal cancer
EMT: epithelial–mesenchymal transition
ESCC: esophageal squamous cell carcinoma
EZR-AS1: ezrin antisense RNA 1
FBS: fetal bovine serum
GAPDH: glyceraldehyde-3-phosphate dehydrogenase
HRP: horseradish peroxidase
lncRNA: long non-coding RNA
TBST: Tris-buffered saline Tween
TGF-β: transforming growth factor β
URHC: up-regulated in hepatocellular carcinoma
